# Native Gating Behavior of Ion Channels in Neurons with Null-Deviation Modeling

**DOI:** 10.1371/journal.pone.0077105

**Published:** 2013-10-25

**Authors:** Wei Wang, Jie Luo, Panpan Hou, Yimei Yang, Feng Xiao, Ming Yuchi, Anlian Qu, Luyang Wang, Jiuping Ding

**Affiliations:** 1 Key Laboratory of Molecular Biophysics of the Ministry of Education, College of Life Science and Technology, Huazhong University of Science and Technology, Wuhan, Hubei, China; 2 School of Engineering, Sun Yat-Sen University, Guangzhou, Guangdong, China; 3 Program in Neurosciences and Mental Health, SickKids Research Institute and Department of Physiology, University of Toronto, Toronto, Ontario, Canada; 4 Key Laboratory of Image Processing and Intelligent Control of Ministry of Education, Department of Biomedical Engineering, College of Life Science and Technology, Huazhong University of Science and Technology, Wuhan, Hubei, China; University of Maribor, Slovenia

## Abstract

Computational modeling has emerged as an indispensable approach to resolve and predict the intricate interplay among the many ion channels underlying neuronal excitability. However, simulation results using the classic formula-based Hodgkin-Huxley (H-H) model or the superior Markov kinetic model of ion channels often deviate significantly from native cellular signals despite using carefully measured parameters. Here we found that the filters of patch-clamp amplifier not only delayed the signals, but also introduced ringing, and that the residual series resistance in experiments altered the command voltages, which had never been fully eliminated by improving the amplifier itself. To remove all the above errors, a virtual device with the parameters exactly same to that of amplifier was introduced into Markov kinetic modeling so as to establish a null-deviation model. We demonstrate that our novel null-deviation approach fully restores the native gating-kinetics of ion-channels with the data recorded at any condition, and predicts spike waveform and firing patterns clearly distinctive from those without correction.

## Introduction

Ion channels are crucial membrane pore proteins that control ionic flows through membrane by opening and closing their gates in response to ligand binding, transmembrane voltage and tension, and generate electric signals that mediate a broad spectrum of biological functions and pathological diseases. Pioneering work by Hodgkin and Huxley [1] provided the first set of voltage-clamp recordings of ion channels at the giant squid nerve axon and ingeniously described the gating kinetics of two voltage-gated ion channels underlying the generation of an action potential with simple mathematic formulas (i.e. H-H Model) [1]. Emerging evidence from both functional and structural studies, however, suggests that ion channels operate using much more complex gating states and transition schemes than simply open and closed states as described by the classical H-H model. Intricate interplay and reciprocal dependence among many ion channels expressed within single neurons further complicate mechanistic analyses of their gating behavior and individual contributions to the neuronal excitability, spike generation and firing patterns. The Markov model, typically composed of multiple “nonconducting” closed states before reaching the “conducting” open state and definable transitional rate constants between any two given states [2]–[5], has overcome the deficits of H-H model and significantly advanced our ability to accurately depict the kinetics and function of ion channels [4] and to predict, simulate, and analyze the dynamics of ion channels governing action potential firings in neurons.

With the growing computational capacity and advanced numerical algorithms to enable automatic fittings of data, H-H or Markov modeling has become increasingly popular, as it allows us to directly build and vary kinetic transition schemes of many gating states for any given channel and to efficiently estimate rate constants from fits to macroscopic ion channel currents acquired with conventional patch-clamp amplifiers. The assumption is that these recorded macroscopic currents mirror channel gating behavior in native neurons with high-fidelity, yet one of the most common problems of automatic fitting is a temporal mismatch between fits and recorded data, raising the possibility that there may be inherited but unidentified errors in the recording system. Indeed, it has been largely ignored that on- and off-line filters in the recording device may introduce significant errors to the size and kinetics of recorded currents. Furthermore, the recorded cell itself can act as a powerful RC filter, particularly under the condition that currents are very fast and large while uncompensated residual series resistance remains substantial in either whole-cell or perforated patch configuration. These inevitable errors, if not taken into careful consideration, may potentially change kinetic properties and confer simulation results totally deviated from native gating behavior of ion channels. More importantly, when multiple channels are integrated into a single model cell to study action potential firing patterns, cumulative kinetic errors from individual channel may exacerbate the convergence of physiological phenotypes between model cell and native neurons.

With the aforementioned issues in mind, we investigated the origins of the mismatch between fits and data, and discovered intrinsic stimulus-response delays at the moment of voltage transition due to the build-in filters of conventional patch-clamp amplifiers as well as residual series resistance (*R*_s_res_) [6], [7]. Given that neither this delay nor *R*_s_res_ is avoidable in real experiments, we developed a null-deviation model to fully correct these errors and reinstate accurate gating kinetics of individual channels in native neurons, and further demonstrated significant changes in the spike waveform and repetitive firing patterns in the null-deviation model cell with multiple channels, which cannot be improved by new technologies, e.g. dynamic clamp [8]. The null-deviation algorithms have been incorporated into the software CeL (www.HustCeL.com) readily available for downloads and applications.

## Results

### Mismatch between recorded data and kinetic fits

**Figure 1:**
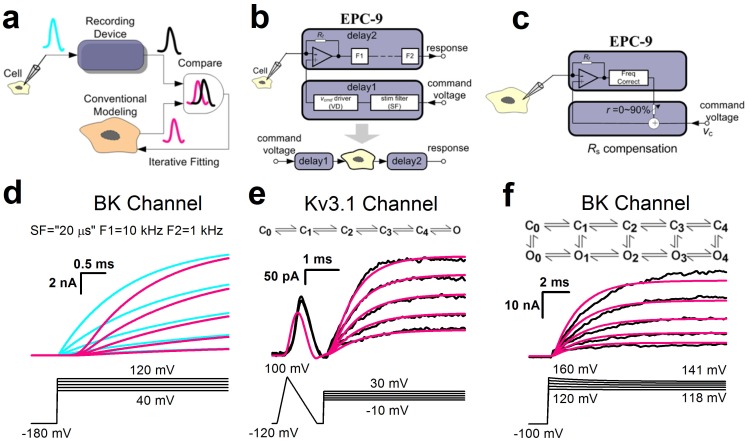
Origin of measurement errors. (a) Schematic diagrams for conventional kinetic modeling. The black signal is data and the purple one is simulation. The cyan signal represents the native signal in a cell. (**b**) Diagram for illustrating the filter effect of an amplifier. For the EPC-9 amplifier, it has several cascaded filters in the open-loop pathway, such as stim filter (SF), filter1 (F1) and filter2 (F2). (**c**) Diagram for illustrating the close-loop *R*_s_ compensation. (**d**) The native traces (cyan) from a BK channel model were stimulated by a voltage command shown at below. The purple traces were acquired from the above cyan traces treated by three filters of a virtual device: SF, F1 and F2. Voltage command is placed at the bottom. (**e**) Top is a 6-state sequential Markov model for Kv3.1 channel; bottom, the black current traces are the data recorded at a lower filtering frequency from MultiClamp 700B, and the purple lines are simulations by a direct fit. Voltage command is placed at the bottom. (**f**) Top is a 10-state Markov model for BK channel; bottom, the black current traces are the data recorded with 90% series resistance (*R_s_*) compensation from an EPC-9 amplifier, and the purple lines are simulations by an F-native fit. At the bottom, the voltage command shows the variable voltages practically applied to BK channel during an experiment with a 90% series resistant compensation.

Numerical automatic or semi-automatic simulation techniques typically require construction of conventional kinetic models that produce the best fits to the experimental data recorded by a patch-clamp amplifier. Here we noted that a direct or conventional fit often produces systematic mismatch with recorded data, being consistently earlier in the temporal onset of activation regardless of the kinetic models used (Figure 1a). We postulated that the deviation of fits from experimental data might originate from the patch-clamp recording system itself and is likely inevitable in real experiments.

We first considered recording device in which two potential delay steps may exist: Delay-1 with a stimulus filter (SF) and Delay-2 with F1 and F2 filters from voltage command to response in the EPC-9 amplifier (or any other amplifier)(Figure 1b). The SF has a filter-dependent effect on the voltage command and the F1 and F2 on recorded signals directly. This phase shift caused by filters in the frequency-domain induces a signal delay in the time-domain. In the EPC-9, Filter2 (F2) is the dominant delay factor; hence, the filter mainly delays cellular responses. The higher we set the F2 cut-off frequency, the smaller delay we get in the response (Figure S1a). Note that most of experiment data in the majority of publications had been collected far below 10 kHz, and hence contained significant inherited errors that were not previously taken into account for kinetic modeling.

Apart from the device filter effects, another source of errors is originated from incomplete series resistance (*R*_s_) compensation. Because it is practically impossible to fully compensate for *R_s_* under experimental conditions, we reasoned that the residual *R*_s_ (or *R*_s_res_) may influence the bandwidth of the command and hence distort the voltage applied to the membrane sufficiently to account for the mismatch in both magnitude and time course (Figure 1c).

When a putative native signal (cyan) from a kinetic model suffers modifications by the multiple-filters of devices, it produces a recording signal (purple) with a significant change including both the time delay and distortion in waveform as shown in Figure 1d. For example, fitting with a 6-state sequential kinetic scheme, we found that the fast-activating Kv3.1 currents (black) in an outside-out patch from recombinant channels expressed in CHO cells were far behind the fits (purple) (Figure 1e). Moreover, the BK currents (black) in whole-cell mode from HEK cells showed the slower activation time courses due to the residual *R*_s_, compared with the fits (purple) from a 10-state rectangular kinetic model (Figure 1f). Taken together, we suggest that both the command voltage and cellular response are inevitably influenced by the recording device, and neglecting these embedded artifacts can lead to contentious results to describe gating kinetics of ion channels in general.

### Virtual device construct and implementation to rectify the mismatch

**Figure 2:**
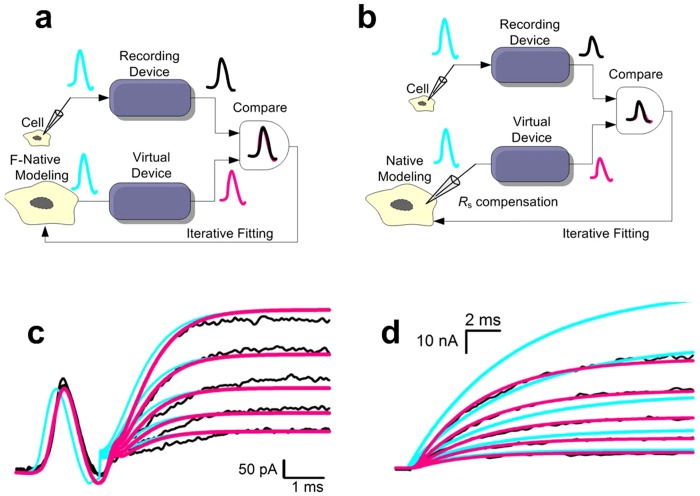
Illustration for principle of native modeling. (a) Schematic diagram for the F-native kinetic modeling. A virtual device mimicking the effect of filters in the patch-clamp amplifier is included in the fitting process. (**b**) Schematic diagram for the F/R-native kinetic modeling. Both the effects of the series resistance and filter are taken into account. (**c–d**) F-native modeling of Kv3.1 channel is shown in (**c**). F/R-native modeling of BK channel is shown in (**d**). The black currents are the data; the purple ones are the simulations by an F-native or a F/R-native fit; the cyan lines are the native responses recreated by the native model. Voltage commands are same to that shown in Figure 1e–1f, respectively.

To overcome the above unavoidable problems, we constructed a virtual device, complying with the same process of collecting and filtering data. This virtual device overcomes the amplifier-dependent deviation in the modeling by: 1) simulating the behavior of various filters in the open-loop signal pathway, termed F-native modeling (or F-native fit) (Figure 2a); 2) mimicking the function of the closed-loop *R*_s_ compensation, termed R-native modeling (or R-native fit) (Figure 2b).

In the open-looped main signal pathway, the stimuli are filtered before adding them to the cell, and the responses are also filtered before sampling using a data acquisition board. We separately modeled the filters in the two pathways with transfer functions *H*_sf_ and *H*_rf_, so that the conditioned stimuli (*V*_p_) and response (*V*_pc_) can be expressed as *V*_p_ = *H*_sf_ *V*_c_ and *I*_pc_ = *H*_rf_ *I*_ion_, where *V*_c_ is the ideal command voltage; *I*_ion_ is the native cellular response; *I*_pc_ is the current recorded at the output of the patch-clamp amplifier. In the software CeL, we re-create the filtering effects in the simulation process by implementing *H*_sf_ and *H*_rf_ (Figure 2a, also See the Materials and Methods, Note S1 and Note S2).

The compensation for the voltage drop due to a series resistance (*R*_s_) compensation is often needed in the whole-cell configuration. The incomplete compensation is another error source, which should be eliminated since it not only lowers but also low-pass filters the command voltage. To reproduce the *R*_s_ compensation from an equivalent circuit shown in Figure S1b, we conditioned the voltage added to the pipette (*V*_p_) by *V*_m_ = (*Z*_m_/(*Z*_m_+*R*_s_))*V*_p_, where *V*_m_ is the membrane potential; *Z*_m_ is the equivalent impedance of the membrane; *R*_s_ is the series resistance. Obviously, the effect of *R*_s_ compensation can be realized with digitally filtering the pipette voltage. However, *V*_p_ is influenced by the close-looped *R*_s_ compensation circuit (See the Materials and Methods and Note S3). During native modeling, we developed algorithms embedded in the software (i.e., CeL), which is capable to remove all the filtering effects and to calculate the actual membrane voltages (*V*_m_) during the emulation of *R*_s_ compensation process simultaneously (Fig. 2b, also See Pseudocode S1 and S2).

After taking into account all the electronic influences, we subsequently applied Markov modeling to generate optimal fits to the data. With the F-native fit approach alone (i.e. *R_s_* is negligible for outside-out patches), the simulation using the same kinetic model for the same date set of Kv3.1 currents shows significant improvements for matching fits with recorded data in both amplitude and time course (Figure 2c). With the F- and R-native fit approach, both filtering effects and residual *R_s_* (for BK channel only) were corrected (Figure 2d). The cyan lines from these models represent native currents of these channels, clearly demonstrating a significant temporal shift in the activation onset (Figure 2c) and an amplitude recovery (Figure 2d) of these channels. Hence, by building a virtual device, we provided an approach for recover the true kinetics of ion channels from experimental results without filter- and residual *R_s_*-dependent distortions.

### Implementating virtual device for kinetic modeling

**Figure 3:**
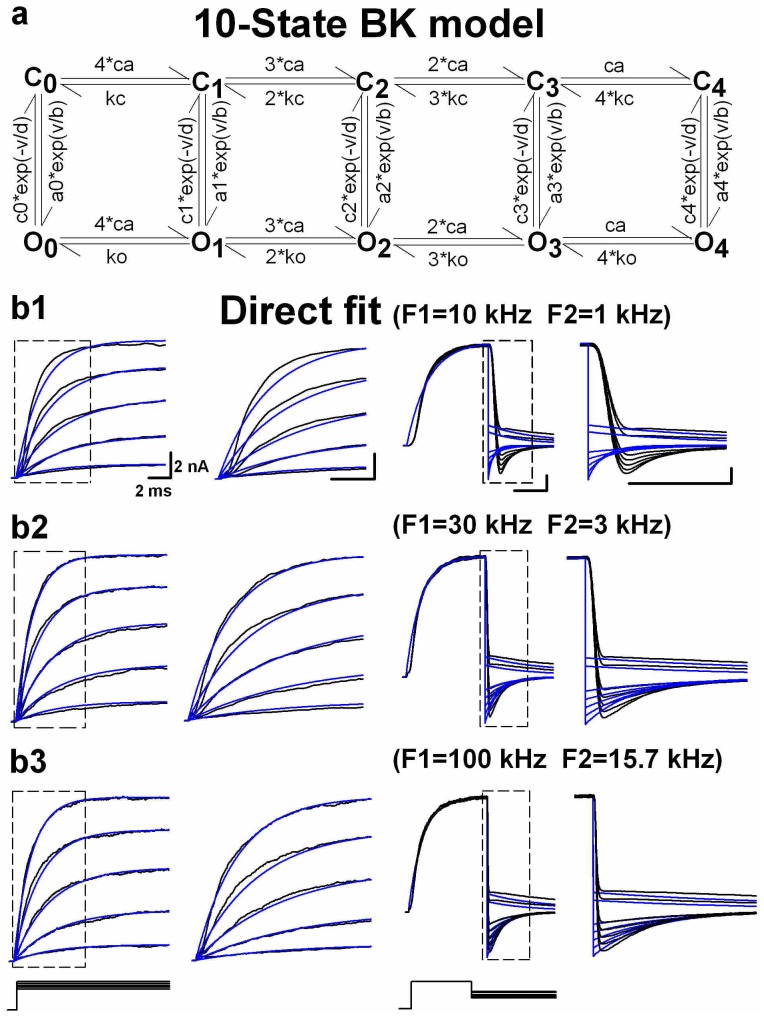
Examples of direct-native or conventional modeling. (a) The 10-state Markov kinetic model of larger-conductance Calcium-activated K^+^ (BK) channels. The letters C and O denote the closed and open states, respectively. The ca and v are [Ca^2+^]_i_ in µM and voltage in mV, respectively. All the parameters of BK models are listed in Table S1. (**b1–b3**) The conventional modeling was applied to the macroscopic BK currents recorded respectively at F1 = 10 kHz, F2 = 1 kHz (**b1**), F1 = 30 kHz, F2 = 3 kHz (**b2**), F1 = 100 kHz, F2 = 15.7 kHz (**b3**) and filtered with Bessel filters, in the presence of 10 µM Ca^2+^. Compared with the data (black), the direct fits (blue) show an earlier onset and an earlier offset with a quick decay in the deactivation case. An expanded view of the boxed region in the left panel is plotted at the right. The voltage protocols are placed under the corresponding currents.

**Figure 4:**
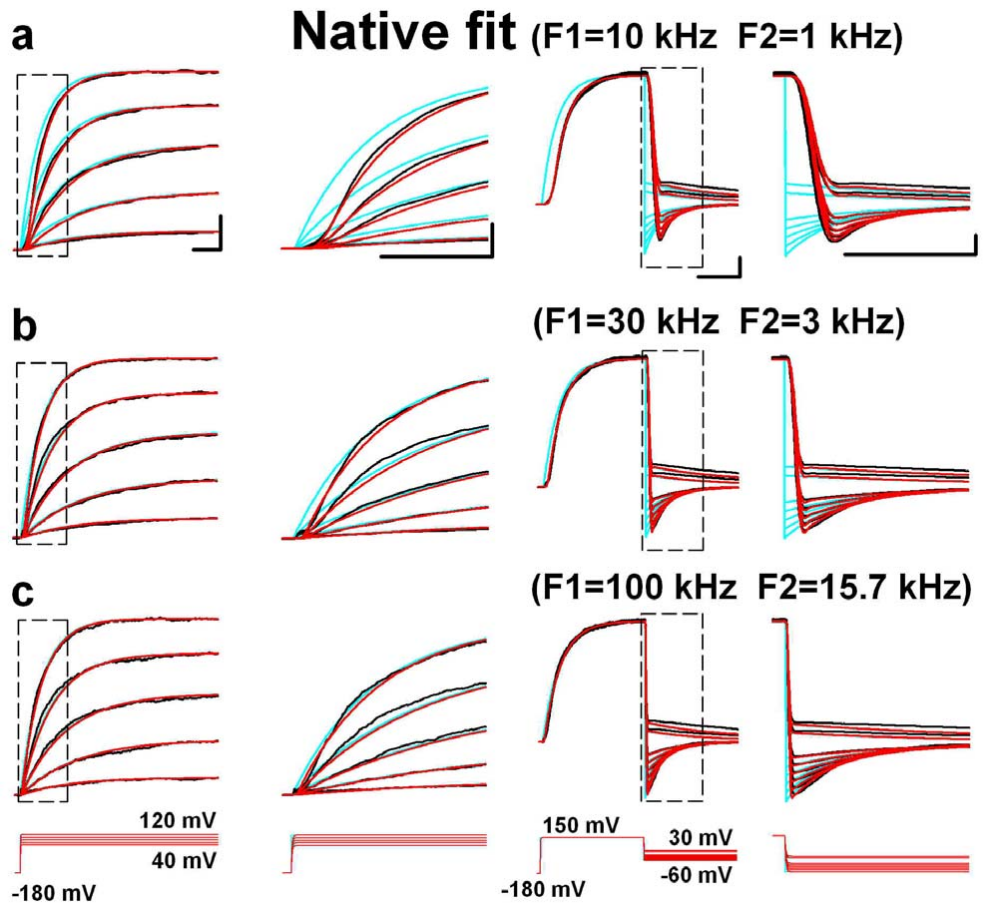
Examples of F-native modeling. (a–c) The F-native modeling applied for BK currents recorded respectively at F1 = 10 kHz, F2 = 1 kHz (a), F1 = 30 kHz, F2 = 3 kHz (**b**), F1 = 100 kHz, F2 = 15.7 kHz (**c**) and filtered with Bessel filters as indicated. The black traces are the data, the red ones the F-native fits, and the cyan ones the postulated inherent traces of currents derived from the F-native model. An expanded view of the boxed region in the left panel is plotted at the right. Both the prototypal (black) and modified (red) voltage protocols are placed under the corresponding currents.

After establishing the effectiveness of our approach to remove artifacts due to the filters and residual *R_s_*, we next simulated and compared kinetic properties of BK channels with and without virtual device. During the simulation of BK channels, a 10-state circular Markov model was used (Figure 3a). However, fitting without virtual device produced a poor match to the data, which was clearly distorted in both the onset and offset (Figure 3b1). In other words, there is a genuine time-delay between data and fits in both the onsets and offsets, caused by the filters in EPC-9 amplifier as we mentioned previously. Obviously, the distortion reduced with increasing filter's frequency (Figure 3b2–b3). For the F-native modeling, we filtered the command voltage mimicking the SF and then the fits mimicking the F1 and F2, based on the recording conditions. Figure 4 shows the good F-native fitting to the data recorded in different Bessel-filtering conditions similar to that in Figure 3b1–b3. Regardless of filtering frequencies for recordings, the rates (or parameters) in three models were virtually the same (Table 1), indicating that the rates of F-native modeling are highly reliable. Even though the current traces recorded in the higher filtering condition (i.e. 100 kHz) seem closer to the F-native current traces (cyan) of channels generated with the virtual device, the F-native fit can always confer a fit better than the conventional fit can even at the higher frequency as it can fully remove all the distortion derived from filters. In contrast, the rates from the direct-fit vary with the filtering conditions significantly (Table 1). Similarly, F-native modeling also conferred good fits to the Butterworth-filtering data recorded with the EPC-9 amplifier (Figure S2). Notably, the Butterworth filter produced the rippled tail currents during deactivation as well as a shoulder that precedes the exponential decay of the deactivation tail current, especially, at lower filtering frequencies, of which both were artifacts. Specially, shoulders were previously considered as a channel reopening event [9], but we demonstrated that they were resulted from both the Bessel and Butterworth filters and can be completely removed by F-native fits (cyan).

**Table 1:** Parameters (Bessel) of BK Model.

	Direct			F-native			
Parameter	1 kHz	3 kHz	15.7 kHz	1 kHz	3 kHz	15.7 kHz	
**a4 (ms^−1^)**	0.07305	0.076650	0.074321	0.067809	0.061997	0.070511	
**b (mV)**	53.17295	47.81222	46.77034	42.36822	42.24442	44.43735	
**c4 (ms^−1^)**	**133.4624**	85.47314	98.14876	110.605	86.97185	105.1186	
**d (mV)**	20.17556	21.28218	22.67607	20.89471	21.01910	21.89965	
**c3 (ms^−1^)**	***0.01242***	***0.026365***	**0.000052**	0.000700	0.000487	0.000499	123 <25%
**c2 (ms^−1^)**	***0.001765***	***0.007818***	***0.000359***	0.000074	0.000087	0.000074	123 <50%
**c1 (ms^−1^)**	***0.010145***	***0.013346***	***0.057316***	0.000551	0.000506	0.000406	**123**<100%
**c0 (ms^−1^)**	***3.80043***	0.162256	**0.031019**	0.212482	0.139127	0.152026	***123***≥100%

kc = 1.17 µM, ko = 19.52 µM for all.

**Figure 5:**
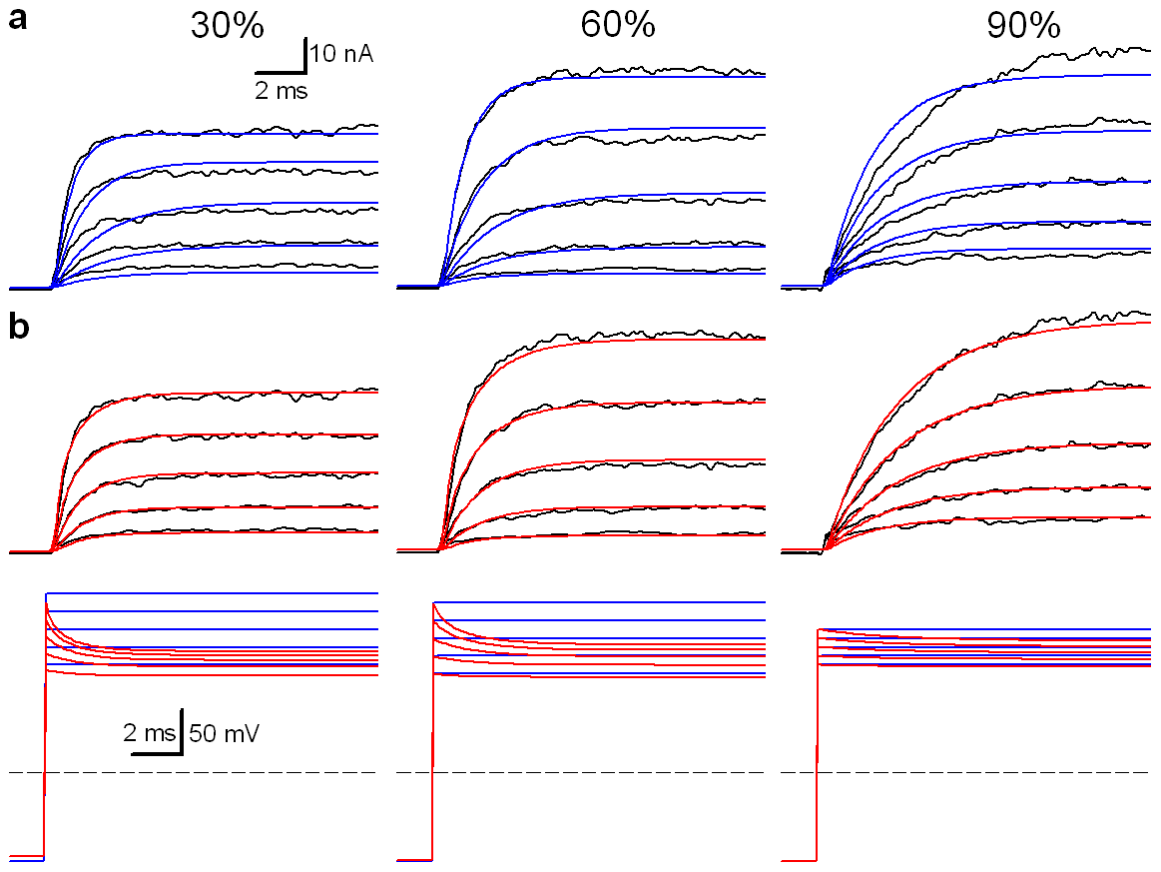
Examples of F/R-native modeling. (a) the F-native (blue) modeling was applied to the BK currents (black) recorded in whole-cell mode in symmetrical 160 K^+^ solutions at 0 Ca^2+^, filtered at 1.2 kHz with *R_s_* = 4 MΩ and *C*_m_ = 12 pF and compensated with 0%, 60% and 90%, respectively. (**b**) Top, the F/R-native (red) modeling was applied to the same BK currents. Bottom, the modified (red) voltage protocols for the F-native (blue) or F/R-native (red) modeling are placed under the corresponding currents.

When the currents are larger, the values of the series resistance (*R_s_*) are extremely critical during recordings, as the actual membrane potentials may be tens of millivolts less than the command voltages, leading to much smaller responding currents. For instance, the whole-cell current of BK channels can be up to ∼100 nA in the whole-cell mode, which is definitely necessary to do the *R_s_* compensation of 80–90% to reduce the loss of voltages, even though the value of *R_s_* is typically around 2 MΩ. However, it is impossible to substantially reduce the loss of voltages by the *R_s_* compensation of even 95%. Specially, the *R_s_* is often more than 10 MΩ in the case of perforated patches, which causes a large error in membrane potentials. With the F/R-native modeling, however, those data can still be used for kinetic fitting work, as exemplified by the recording of BK currents in Figure 3a. The activation currents of BK channels (black) were recorded in the whole cell mode with the *R_s_* compensation of 30% (left), 60% (middle), and 90% (right), respectively. The 10-state BK model was used for the global F-native fitting to all the current traces of BK channels (Figure 5a), which seems a good but not perfect fit. The major problem is that their voltage-dependent time constants show an apparent deviation from those of data, because the actual stimulating voltage is much lower than that used in F-native fits. Furthermore, the *R*_s_res_ still exists even after the optimal compensation, and alters the command voltages applied to the membrane (Figure 5b, bottom). In one of the cases of 90% compensation (i.e. *r* = 90%), the whole-cell membrane resistance *R*_m_ was 1/(NOC*p_o_*G_BK_) = 4.7 MΩ at 160 mV, where *p*_o_ is the open probability of channels, NOC the number of channels and *G_BK_* the single channel conductance of BK. Given *R*_s_ = 4 MΩ, we can calculate the actual stimulus voltage on membrane; i.e., *v*_m_ = (*R*_m_/(*R*_m_+(1−*r*)*R*_s_))*v*_c_ = 92%×160 = 147 mV, consistent with that predicted by native modeling (Table S1). In addition to the F-native modeling, we performed the R-native modeling by a virtual *R*_s_ compensation (i.e. F/R-native modeling) prior to fitting. Not only did the F/R-native modeling confer a much better fitness than the F-native modeling (Figure 5b, top), but it also predicted the actual membrane voltage under arbitrary compensation (Figure 5b, bottom). Clearly, two sets of parameters generated by two methods are quite different in the same modeling, because the F/R-native fit uses the voltage command corrected, but the F-native fit uses the one pre-designed (Figure 5b, bottom). Even though the best fitness belongs to the 90% compensated data, it still shows a clear difference to the native (cyan) traces (Figure 2d), indicating that implement this virtual device is necessary to rectify errors associated with real experiments.

### Physiological consequence of model cell integrated with native modeling

**Figure 6:**
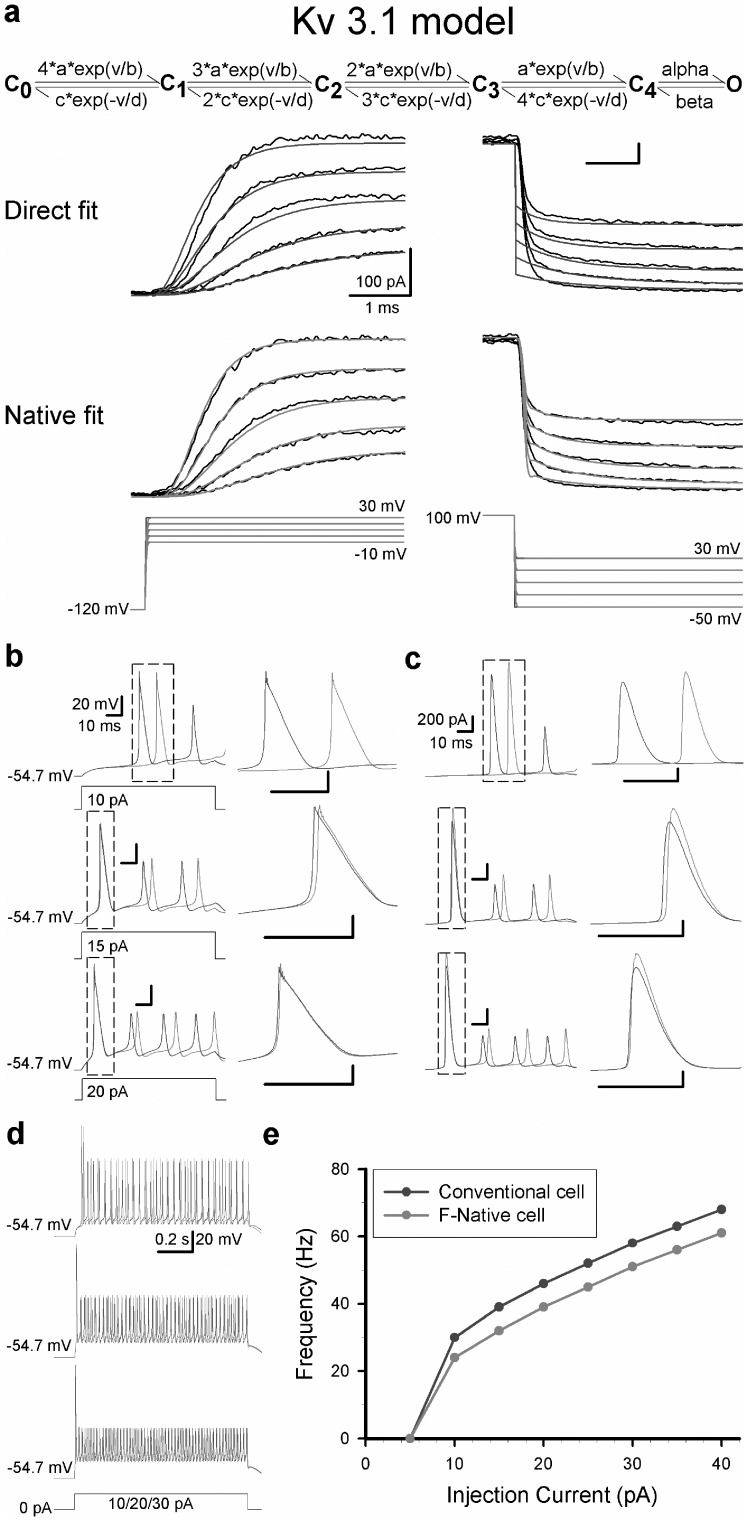
Comparison of action potentials (APs) in a model cell composed of Nav and Kv3.1 channels. (a) Top, the 6-state Markov kinetic model of the fast activated K^+^ (Kv3.1) channels. Here the letters C_i_ (i = 0, 1, 2, 3 and 4) and O denote the closed and open states, respectively. In this model, the letters a, c, alpha and beta are rate in ms^−1^ and b, d and v are voltage in mV, respectively. Middle, the direct (blue) modeling was applied to the Kv3.1 currents (black) of both the activation (left) and deactivation (right) recorded in an outside-out patch at 4 kHz. Bottom, the F-native (red) modeling was applied to the Kv3.1 currents (black) of both the activation (left) and deactivation (right) recorded at 4 kHz. Both the prototypal (blue) and modified (red) voltage protocols are placed under the corresponding currents. All the parameters of Kv3.1 models are listed in Table S2. (**b**) Left column, the repetitive AP firings evoked respectively by an injected current of 10 pA (top), 15 pA (middle) and 20 pA (bottom) for 100 ms as indicated. The red APs come from a model cell containing a model Kv3.1 channel produced by F-native modeling and the blue ones from a model cell containing a model Kv3.1 channel produced by conventional modeling. Inbox shows the first APs. Right column, a comparison of the first AP derived from the conventional (blue) and F-native (red) models, respectively. (**c**) Left column, a comparison of the Kv3.1 currents during the above APs. The red trace is for the F-native cells and the blue one is for conventional cells. Inbox shows the currents of the first APs. Right column, a comparison of the first Kv3.1 currents during the first AP derived from the conventional (blue) and F-native (red) cells, respectively. The corresponding parameters of two model cells are listed in Table S3. (**d**) Three AP's firing patterns were produced by 1 s-duration injection currents of 10/20/30 pA, respectively. The red lines are for the F-native cells, the blue line for conventional cells. (**e**) The spike numbers were plotted as function of injection currents. All the AP's spikes counted here must have a peak value of over −20 mV.

To evaluate the significance of the native modeling, we built a minimal model cell that was composed of a sodium channel (Nav), a background leak channel [4] and Kv3.1 channels with or without correction by F-native modeling based on the activation and deactivation parameters of Kv3.1 currents fit with the 6-state kinetic model (Figure 6a). We noted a difference of ∼50% from the exponential factor *d* of deactivation between the parameters of the conventional and native fit. Moreover, all the forward pre-exponential parameters of activation are ∼10% larger than that of backward ones (Table S2). When injecting a depolarizing current of 10 pA, the model cell containing the Kv3.1 channels with conventional modeling (blue) produced only 1 AP, occurring much earlier than the spike in the model cell with the F-native modeling (red) in a duration of 100 ms (Figure 6b top) (Table S3). This predicts that their firing frequencies of APs will be significantly different. Comparing the first AP of the conventional model cell, the F-native model cell showed a clear delay (Figure 6b top right) due to the larger Kv3.1 currents in the F-native cells (Figure 6c). The temporal onset of subsequent APs in the two cells were gradually shifted apart after the first AP, (Figure 6b), indicating that a subtle difference in channel kinetics can be manifested into very larger difference in the waveforms and firing patterns of APs. With increasing level of the injected current (i.e. 2 s), the number of evoked spikes of two model cells increases but diverges in trajectory significantly, giving entirely different input-output relationships (Figure 6d–6e). Because a cell usually contains variety of different channels, one can envisage that subtle errors of each channel in kinetics can carry over into the model cell and cause significant changes in AP firing patterns. These results demonstrated that the null-deviation kinetic modeling can effectively rectify unavoidable errors intrinsic to the recording system, and simulate and predict physiological functions of ion channels and the excitability of neurons at an unprecedented accuracy and reliability.

## Discussion

This distortion and improvement of experimental recording have been reported by several laboratories [6], [7]. However, they are concentrating on the errors in current clamp. In contrast, we are concerning on the mismatch between the data and kinetic model in voltage clamp. Using the approach presented in this study, we can get the native (or null-deviation) kinetic modeling for all kinds of channels with those data recorded even at very low frequency, which cannot provided simply by infinitely increasing recording frequency or improving amplifier such as dynamic clamp and so on [8].

Computational modeling has emerged as one of the most important new frontiers of neuroscience, particularly for addressing burning issues in complex systems that cannot be readily studied with traditional deduction approaches in experiments. In this regard, kinetic modeling of ion channels becomes an indispensable approach to understand intricate interactions among more than a dozen of ion channels that single central neuron typically employs to regulate its excitability and generate heterogeneous firing patterns encoding a wide spectrum of information. Unfortunately, much of previous work in this area may carry significant errors or even completely flawed as a result of inherited device errors. In this study, we have revealed a series of embedded errors associated with patch-clamp technique, which ultimately cause mismatch between the data and fits during automatic fitting of conventional kinetic modeling. To resolve these inevitable problems originated from real experiments, we have developed a novel approach, namely native (or null-deviation) kinetic modeling which has significant utility for bridging the gap between experimental recordings and computer simulations of ion channels in general.

With the assumption that the patch-clamp recordings can perfectly mirror the channel activities, all the jumps and fluctuations in traces are considered biologically originated. In contrast, we have demonstrated that various filters built in conventional amplifiers also contributed to waveform shaping; e.g., the Bessel type filter sloped the vertical edges (Figure 4), and the Butterworth type filter introduced ripples (Figure S2). Therefore, we fully removed the filter effects by introducing the F-native modeling. As the filtering frequency increased, the experimental traces and the fits gradually went toward the F-native traces (Figure 4, Figure 2), suggesting that the instantaneous responses of channels possessed vertical edges, accurately represented by the F-native traces. However, the instantaneous events, like the vertical deactivation of BK channel, could never be recorded with a finite bandwidth recording device, and it could only be optimally investigated by filtering with a higher frequency, which, however, means large background noise. For a fast activating channel like Kv3.1, a small delay, e.g. 0.1 ms, caused by the common used bandwidth (4 kHz) would be unbearable (Figure 1e). In case of ultra-fast channel activities, which possessed the comparable time constants to that of the highest filtering frequency, the “instantaneous” events could never be recorded directly. Therefore, the F-native modeling is probably the only way to investigate the “instantaneous” events.

The impact of residual *R*_s_ with compensation rate >85% is usually neglected in patch-clamp experiments. We found that, in the case of larger currents, a small residual *R*_s_ could decrease the archetypal voltages up to ten of millivolts, and that it would also contribute to significant mismatch in time and size of simulated and experimental results if not corrected, particularly when currents are ultra fast and large. To address this issue, we have proposed the R-native modeling, which implemented a virtual *R*_s_ compensation during fitting. In most of the time, the R-native modeling should be used in combination with the F-native modeling. We have demonstrated that a combined F-and R-native modeling with our virtual device could effectively eliminate the device effect off-line and produce the null-deviation simulations of macroscopic currents recorded under non-idealistic experimental conditions. With the F- and R-native modeling, one can set the hardware to any bandwidth and *R*_s_ compensation rate, and the software built-in with virtual device will incorporate these experimental variables into simulation and reinstate the native response of channels. In this way, the native modeling differentiates the biological and electronic contributions in the recorded currents.

Finally, we have demonstrated dramatic differences of the waveform and firing pattern between the two-channel model cells with and without the virtual device for correcting one of channels (Figure 6), indicating that the corrections are necessary for all the channels integrated in a typical central neuron to accurately generate various physiological phenotypes.

For the moment, the native modeling generates the best fit to data and produces a null-deviation simulation of ion channels. To account for the filter effects, some other strategies, apart from the virtual device emulation strategy, could be used, and the most common one is to inverse-filter the recorded currents [10]. The basic idea of the inverse-filtering is to undo the filtering effect by sending the recorded current to a filter with inverse effect to the filters in the amplifier. However, this technique is less practical for three reasons: first, it amplifies the high-frequency noise; second, the current reinstating is very sensitive to parameters of the inverse filter; third, the overall bandwidth of the combined filter and inverse filter is still finite. In other words, the restored data at the best is only an approximation of the response but not the native one.

Instead of recovering the native response precisely, another strategy is to take account of just the signal delay. One method falls in this category is the delayed fit. The delayed fit is a method of delaying the command voltage by inserting a segment of reference potential (e.g., the holding potential) while fitting, so that the onset/offset of the fit and data are aligned. To generate good fitness, the delayed intervals for different filtering frequency cases were varied (Figure S3). The higher the filtering frequency, the smaller the delayed interval. We found that the optimal delayed interval at different frequency was consistent with the calculated EPC-9 total delay (Table 2, Figure S1a, Note S1), suggesting again that the inherent filters in the patch-clamp amplifier caused signal delay. In spite of this, the delayed fit could never, however, generate a declining deactivation similar to that of experimental data (Figure S3), and the fitting quality, denoted by the parameter consistency, of the delayed fit was between the direct fit and F-native fit (Table 1, Table S4). One can also delete a segment of experimental trace and then make a fit, which is termed as the deleted fit. The deleted fit aligned the onset/offset as the delayed fit. However, this approach changed the length of the recording and would induce other problems, such as time disorders.

**Table 2:** Signal delay caused by EPC-9 (stim. filter: 20 µs; *R_f_* = 50 GΩ).

	*t*_delay_ (ms)			
	Bessel			
*f*_F2_3dB_ (kHz)	F1: 10 kHz	F1: 30 kHz	F1: 100 kHz	F1: High Q
**1**	0.634	0.615	0.609	0.614
**2**	0.385	0.366	0.361	0.365
**3**	0.301	0.283	0.277	0.282
**4**	0.260	0.241	0.235	0.240
**5**	0.234	0.216	0.210	0.215
**6**	0.218	0.199	0.193	0.198
**7**	0.206	0.187	0.181	0.186
**8**	0.197	0.178	0.172	0.177
**9**	0.190	0.171	0.165	0.170
**10**	0.184	0.165	0.160	0.164
**11**	0.180	0.161	0.155	0.160
**12**	0.176	0.157	0.152	0.156
**13**	0.173	0.154	0.148	0.153
**14**	0.170	0.151	0.146	0.150
**15**	0.167	0.149	0.143	0.148
**16**	0.165	0.147	0.141	0.146

Our method also has a good generality. The parameters of the virtual device were defined based on the design scheme of the device (Figure 1b and 1c). The values of these parameters of our virtual device were set according to hardware configuration, which were restored in files as well as data. Since most of the patch-clamp amplifiers were designed under a common scheme, the virtual device of our method was of good generality. Further, we actually provided two strategies for HEKA and Axon amplifiers, respectively. The CeL software can recognize the data format and choose a proper correct scheme to generate the most accurate model.

In conclusion, we suggest that the null-deviation modeling with readily implementable virtual device developed here is both sufficient and necessary to truly depict the gating kinetics of ion channels. The null-deviation modeling will provide a broad utility and mechanistic dimension for the studies of ion channels and their physiological significance in a variety of excitable cells. Therefore, our method is particularly useful to establish the accurate kinetic model for all the ion channels to characterize the neuronal activity in nerve system.

## Materials and Methods

### Cell culture and Transfection

HEK293 cells were cultured in modified Eagle's medium (DMEM, Gibco) supplemented with 10% fetal bovine serum (FBS, Gibco) at 37°C incubator with 5% CO_2_. The day before transfection, cells were transferred into a 24-well plate. Full length cDNA of mSlo1 (Accession NO. NP_034740) was subcloned transiently into pcDNA3.1/Zeo (Invitrogen) using lipofectamine 2000 (Invitrogen) according to manufacturer's protocol. Recordings were carried out one day after transfection. Chinese hamster ovary (CHO) cells with stable expression of Kv3.1 channels were generously provided by Dr. Leonard K Kaczmarek (Yale).

### Electrophysiology

For mSlo1 currents from HEK293 cells, both the inside-out and whole-cell modes were used. For the inside-out mode recording, the bath solution contained the following (in mM): 160 MeSO_3_K, 10 HEPES (pH 7.0), 5 HEDTA with added CaCl_2_ for 10 µM free Ca^2+^, as defined by the EGTAETC program (McCleskey, Vollum Institute, Portland, OR). The pipette solution contained (in mM): 160 MeSO_3_K, 2 MgCl_2_, 10 HEPES (pH 7.0). And for whole-cell mode recording, the bath solution contained the following (in mM): 160 MeSO_3_K, 2 MgCl_2_, 10 HEPES (pH 7.0). The pipette solution contained (in mM): 160 MeSO_3_K, 10 HEPES (pH 7.0), 5 EGTA. While for Kv3.1 currents from CHO cells, the outside-out mode was used. The bath solution contained (in mM): 140 NaCl, 2.5 KCl, 1.3 CaCl_2_, 10 HEPES (pH 7.3), 33 glucose and the intracellular solution included (in mM): 97.5 K-gluconate, 32.5 KCl, 5 EGTA, 40 HEPES (pH 7.3), 1 MgCl_2_. All chemicals were purchased from Sigma (St. Louis, MO). Patch pipettes pulled from borosilicate glass capillaries with resistance of 1.5–6 megohms when filled with pipette solution. Experiments were performed using EPC-9 patch-clamp amplifier (HEKA, Germany) for mSlo1 current recordings, and 700A amplifier (Axon, USA) for Kv3.1. Currents were digitized and filtered as indicated in the figures and the series resistances were compensated as required. All the experiments were performed at room temperature (22–24°C).

### Data Analysis

Data were analyzed with Clampfit (Axon Instruments, Inc.) and Sigmaplot (SPSS, Inc.) software. The conductance-voltage (G-V) curves for activation were fitted to a Boltzmann equation as below, *G/G_max_ = {1+exp[(V−V_50_)/k]}^−1^*, where *V_50_* is the half maximal voltage, *G* the conductance, *G*_max_ the maximum conductance and *k* the slope factor.

### Simulation of the open-loop filter delays

Most of the patch-clamp amplifiers are designed to involve some filters in its open-loop main signal pathway. For example, the EPC-9 amplifier has a stimulus filter (SF) in its stimulus pathway, and the filter1 (F1) and the filter2 (F2) are in its current monitor pathway.

The stimulus filter causes a slight delay in the command voltage before adding to the cell. In most cases, the SF is set to “20 µs”, and we simulate its functionality by low-pass filtering the command voltage using a difference equation
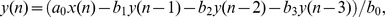
Where, *b*_0_ = 1; *b*_1_ = −2(2*p*+*q*); b_2_ = (*p*_2_+2*pq*); *b*_3_ = −*p* ^2^ *q*; *a*_0_ = *b*_0_+*b*_1_+*b*_2_+*b*_3_; *p* = e^(-*Ts*/*T*10)^; *q* = e^(-*Ts*/*T*2)^; *T*10 = 1×10^−5^; *T*2 = 2×10^−6^; *Ts* is the value of the sampling interval, say 1×10^−5^ at the 100 kHz sampling rate.

In the EPC-9, the filters in the current monitor pathway delays the cellular response. The F1 has four candidates, and the F2 is always designed to have a “Bessel” or “Butterworth” specification [11]. Since the F2 has an influence superior to the F1, we simplified the simulation by ignoring the F1 except when considering the filter order. Specifically, if the F2 is set to have a “Bessel” specification, the overall filter has an order of 6 for the F1 is a second-order low-pass filter, and the F2 is fourth-ordered. However, if the F2 is set to “Butterworth”, the overall filter has only an order of 4 for the F1 is neglected compared with the dominant F2. Both the “Bessel” and “Butterworth” filters are simulated using the standard functions.

In the other patch-clamp amplifiers, the filters in the open-loop pathway are given in the relative datasheets. For example, the Axon amplifier is usually designed to have a fourth-order “Bessel” or “Butterworth” filter. The signal delay caused by SOF (Signal Output Filter) of the AXON type patch-clamp amplifier was shown in Table S5.

### Simulation of the closed-loop *R*_s_ compensation

The principle of the closed-loop *R*_s_ compensation is shown in Figure 1f. To compensate for the voltage dropped across the electrode (i.e., the *R_s_*), the same amount of voltage should be adding to the command voltage; hence, the membrane potential (*v*_m_) is clamped to the command (*v*_c_). The most used scheme is the closed-loop positive feedback method [11]. However, the instability at high voltage clamping bandwidth limits the compensation completeness. Patch-clamp amplifiers usually can only compensate for 90% of the series resistance at most. The pipette current (*i_p_*) is determined by the series resistance (*R*_s_), the transmembrane resistance (*R*_m_) and the membrane capacitance (*C*_m_). The value of *R*_s_ and *C*_m_ is estimated when collecting the experimental data, and the only unknown is the time-and-voltage-variant *R*_m_ when applying the voltage protocols. Fortunately, given the single-channel conductance and number of channels, the value of *R*_m_ (i.e., the reciprocal of the channel conductance when activated) can be predicted in our software CeL. Therefore, to simulate the closed-loop *R_s_* compensation, we used a two step recursive strategy. First, we calculated the real *v*_m_ for a given *R*_m_ at a specific time point using the following formulation:
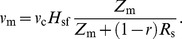



Where, *r* is the compensation rate, ranging from 0 to 90%; *Z*_m_ is the equivalent complex impedance of the paralleled *R*_m_ and *C*_m_; *H*_sf_ is the transform function of the stimulus filter, thus we have
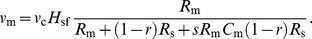



It is obvious that when *r* = 100%, the membrane potential (*v*_m_) equals to command voltage (*v*_c_). Second, we calculated *i*_p_ and *R*_m_ for the next time point.

 According to above equations, the closed-loop *R*_s_ compensation plays a role of a low-pass filter on the command voltage, even if at the highest compensation rate (e.g. 90%).

In this study, all the results were simulated with the software CeL, a useful tool for precisely fitting of model-data (www.HustCeL.com). The CeL has three main modules: (1) Voltage-clamp for kinetic simulation of ion channels [4]; (2) Current-clamp for simulating the APs of cells [4]; (3) Fit-kinetics for automatically calculating the parameters of kinetic models [5]. The software is running on Windows XP. For those who use the CeL to publish any work, authors request the software “CeL (HUST)” specifically referred.

## Supporting Information

Figure S1 **Illustration of device effect.** (a) Total delay caused by the EPC-9 was plotted as a function of the cut-off frequency of F2. (**b**) A schematic diagram of an equivalent circuit for the *R_s_* compensation.
(JPG)

Figure S2 **The Butterworth-filtering effect of EPC-9 amplifier.** All the same as described in Figure 3c **.**
(JPG)

Figure S3 **Comparison between the non-delayed (direct) and delayed fits.** (**a1–a2**) The activation (**a1**) and deactivation (**a2**) currents of BK channels, recorded in F1 = 10 kHz and F2 = 1 kHz, were fitted to the BK model with a delay-time Δt = 0.634 ms (green). No delayed (black) or delayed (green) protocol is placed under the traces. The boxed regions were zoomed in for more details. Trace is black and fit red. (**b1–b2**) The same as described in **a1–a2** except F1 = 30 kHz and F2 = 3 kHz. The delay-time is Δt = 0.283 ms. (**a1–a2**) The same as described in **a1–a2** except F1 = 100 kHz and F2 = 15.7 kHz. The delay-time is Δt = 0.141 ms.
(JPG)

Table S1 **Parameters of BK channel models.**
(DOCX)

Table S2 **Parameters of Kv3.1 channel model.**
(DOCX)

Table S3 **Parameters of the model cell composed of Nav and Kv3.1 channels.**
(DOCX)

Table S4 **Comparison of parameters of BK Model derived by delayed fit (at different bandwidth) and native fit (Bessel, 15.7 kHz).**
(DOCX)

Table S5 **Signal delay caused by SOF of the AXON type patch-clamp amplifier.**
(DOCX)

Note S1 **Calculation of the filter delay caused by the EPC-9.**
(DOCX)

Note S2 **Calculation of the filter delay caused by Axon 700B.**
(DOCX)

Note S3 **Derivation of the relationship between** ***v*** **_c_ and** ***v*** **_m_ in the closed-loop** ***R*** **_s_ comepnsation.**
(DOCX)

Pseudocode S1 **Emulation of Stim Filter.**
(DOCX)

Pseudocode S2 **Emulation of response filter and Rs Compensation.**
(DOCX)
