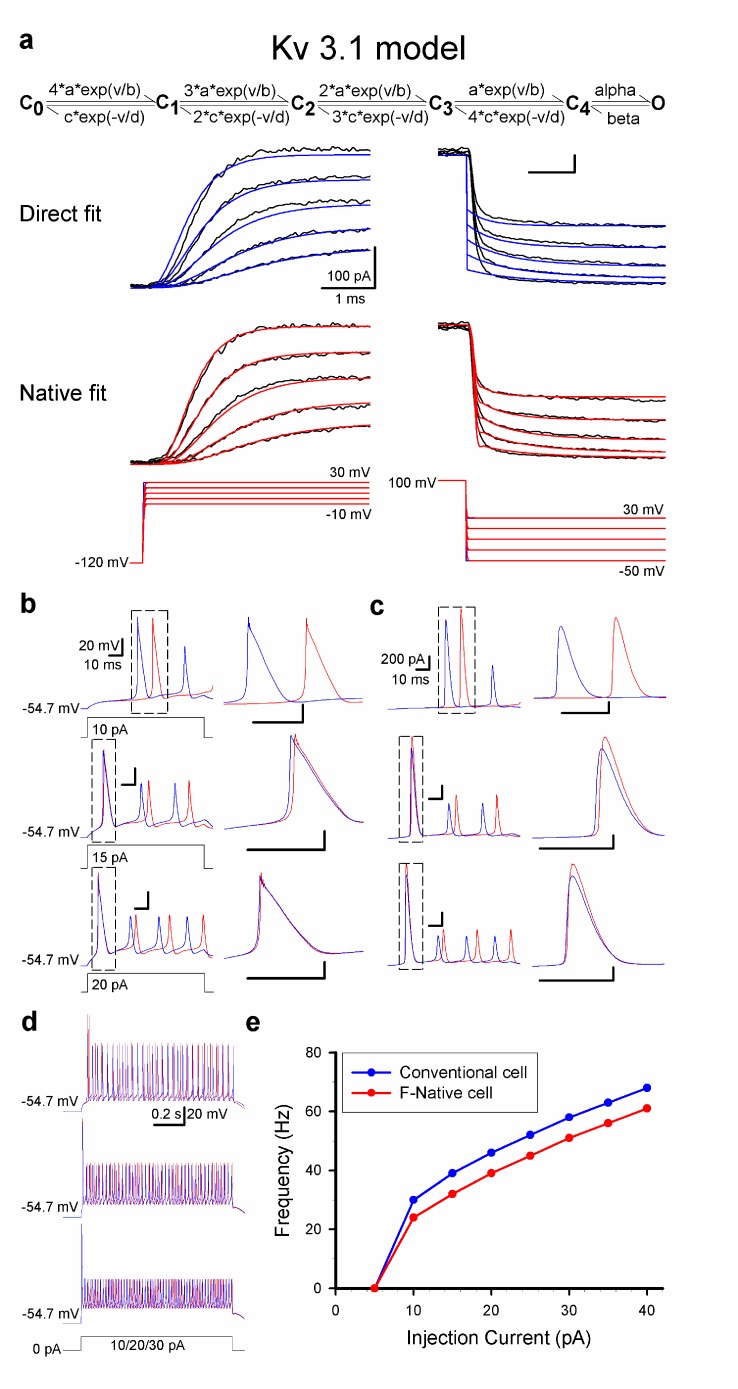# Correction: Native Gating Behavior of Ion Channels in Neurons with Null-Deviation Modeling

**DOI:** 10.1371/annotation/ec003066-bdc5-43f4-bf81-50dcb3ae3459

**Published:** 2013-12-11

**Authors:** Wei Wang, Jie Luo, Panpan Hou, Yimei Yang, Feng Xiao, Ming Yuchi, Anlian Qu, Luyang Wang, Jiuping Ding

Figure 6 was erroneously published without color. Please see the corrected Figure 6 here: 

**Figure:**