# Supplementary figures and images for: Native Gating Behavior of Ion Channels in Neurons with Null-Deviation Modeling
Source: PLoS One. 2013 Oct 25;8(10):e77105. doi: 10.1371/journal.pone.0077105 (PMC3808363; doi:10.1371/journal.pone.0077105)

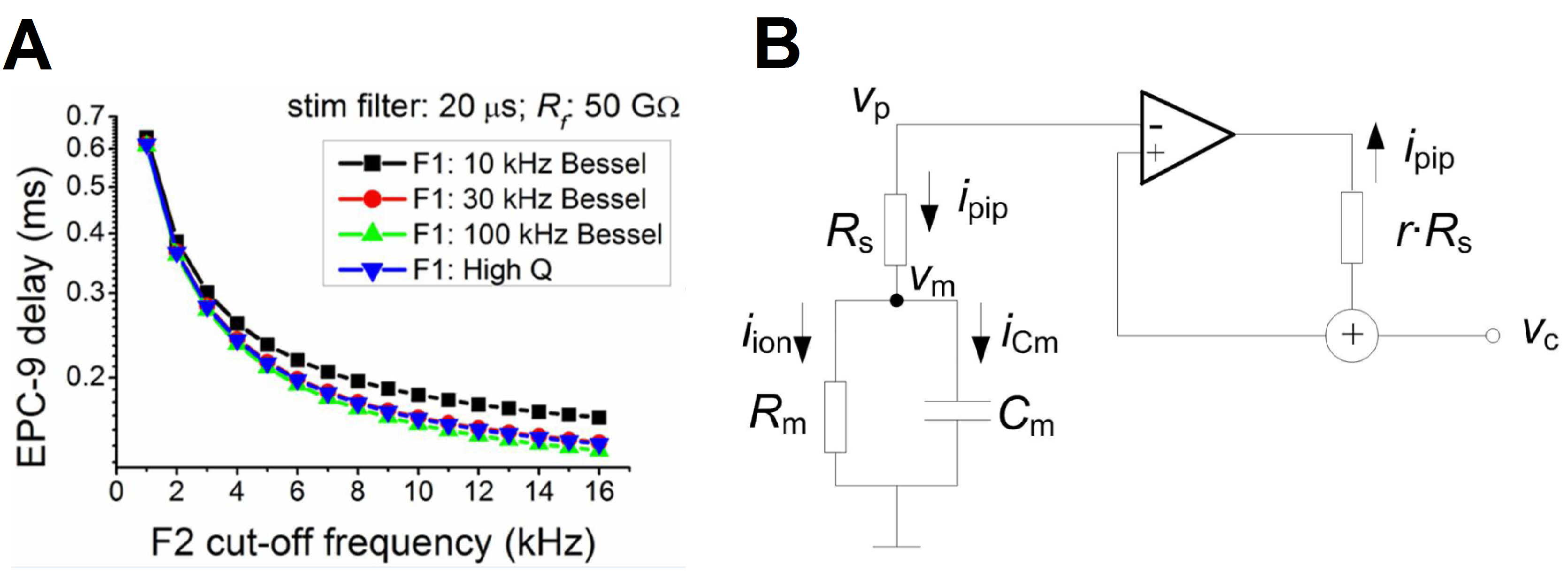

Supplement: Figure S1 — Illustration of device effect. (a) Total delay caused by the EPC-9 was plotted as a function of the cut-off frequency of F2. (b) A schematic diagram of an equivalent circuit for the Rs compensation. (JPG) [file pone.0077105.s001.jpg]

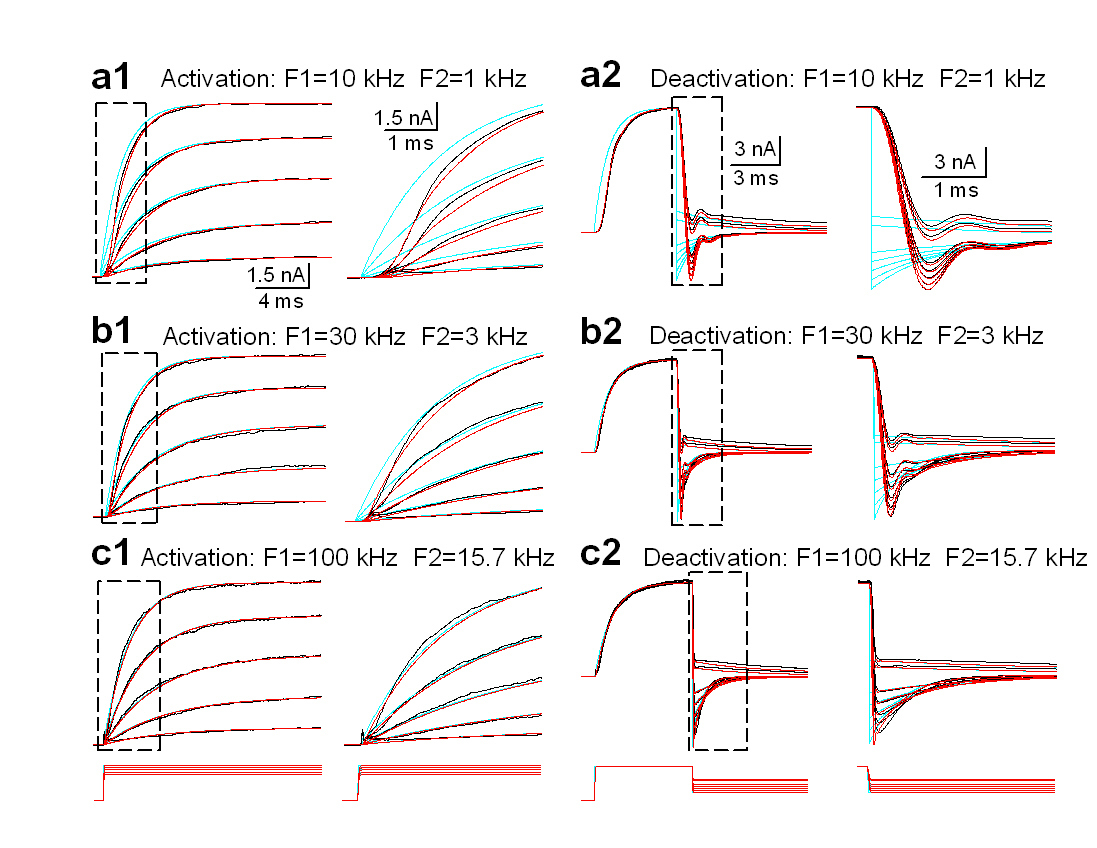

Supplement: Figure S2 — The Butterworth-filtering effect of EPC-9 amplifier. All the same as described in Figure 3c . (JPG) [file pone.0077105.s002.jpg]

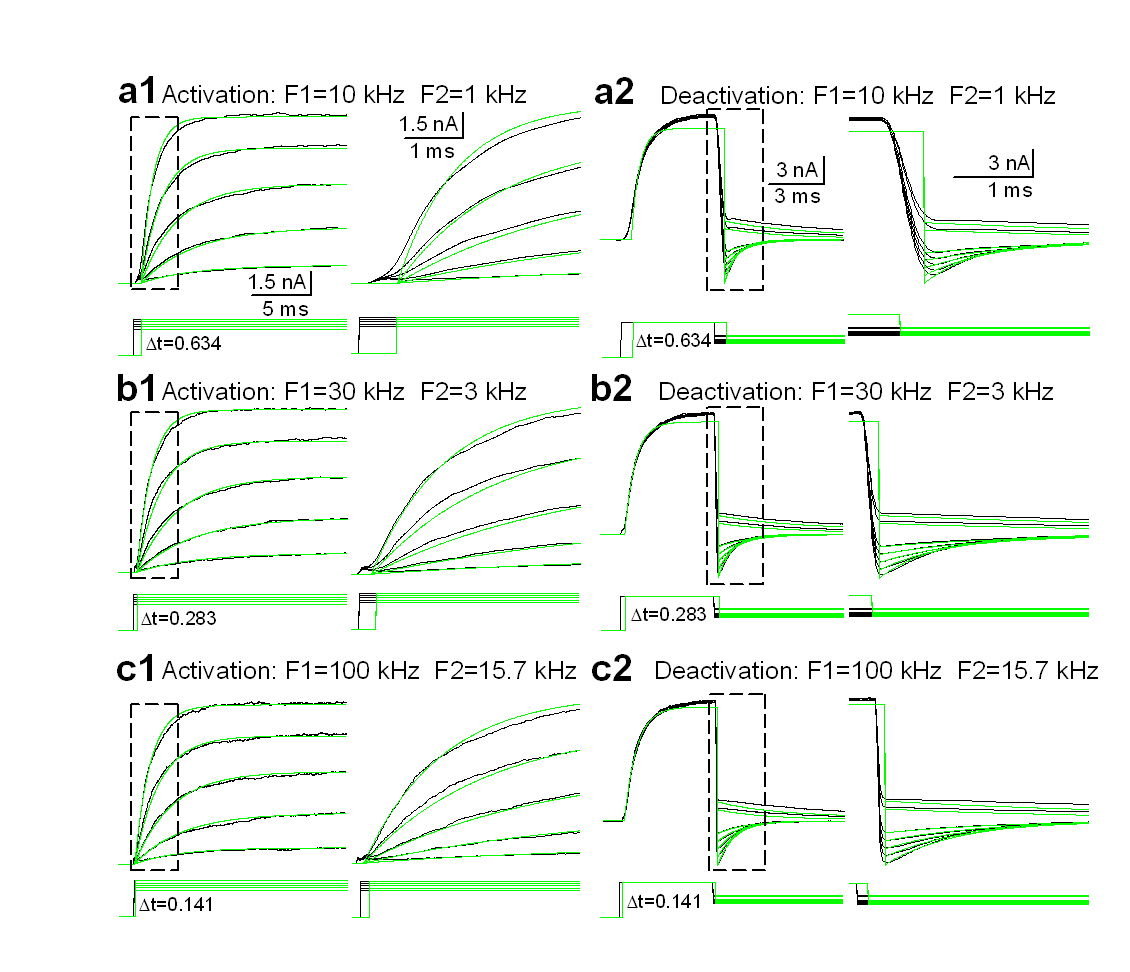

Supplement: Figure S3 — Comparison between the non-delayed (direct) and delayed fits. (a1–a2) The activation (a1) and deactivation (a2) currents of BK channels, recorded in F1 = 10 kHz and F2 = 1 kHz, were fitted to the BK model with a delay-time Δt = 0.634 ms (green). No delayed (black) or delayed (green) protocol is placed under the traces. The boxed regions were zoomed in for more details. Trace is black and fit red. (b1–b2) The same as described in a1–a2 except F1 = 30 kHz and F2 = 3 kHz. The delay-time is Δt = 0.283 ms. (a1–a2) The same as described in a1–a2 except F1 = 100 kHz and F2 = 15.7 kHz. The delay-time is Δt = 0.141 ms. (JPG) [file pone.0077105.s003.jpg]
