# Supplementary material for: Native Gating Behavior of Ion Channels in Neurons with Null-Deviation Modeling
Source: PLoS One. 2013 Oct 25;8(10):e77105. doi: 10.1371/journal.pone.0077105 (PMC3808363; doi:10.1371/journal.pone.0077105)
Supplement: Table S1 — Parameters of BK channel models. (DOCX) [file pone.0077105.s004.docx]

**Table S1. Parameters of BK channel models.**

|  | F-native | | | Native | | |
| --- | --- | --- | --- | --- | --- | --- |
| kc (μM^-1^) | 906.396489 | | | 733.370705 | | |
| ko (μM^-1^) | 138.438951 | | | 67.308941 | | |
| a4 (ms^-1^) | 50.883519 | | | 11.126548 | | |
| b (mV) | 28.866231 | | | 27.599061 | | |
| c4 (ms^-1^) | 181.869338 | | | 104.079656 | | |
| d (mV) | 40.944705 | | | 23.063228 | | |
| c3 (ms^-1^) | 72.692722 | | | 5.409872 | | |
| c2 (ms^-1^) | 318.224924 | | | 15.084666 | | |
| c1 (ms^-1^) | 4.299723 | | | 0.849555 | | |
| c0 (ms^-1^) | 8.776138 | | | 66.183005 | | |
| Filter(Bessel) | 1.2 kHz | | | | | |
| R_s_ | 4 MΩ | | | | | |
| C_m_ | 12 pF | | | | | |
| *r*: NOC | 30%: 451 | 60%: 668 | 90%: 1119 | 30%: 2433 | 60%: 2150 | 90%: 1828 |
| *v*_c_: Predicted *v*_m_ | Cannot predict | | | 160 mV: 125 mV | 160 mV: 133 mV | 160 mV: 147 mV |
| *p*_o_ | 160 mV: 0.66 | 160 mV: 0.66 | 160 mV: 0.66 | 125 mV: 0.22 | 133 mV: 0.30 | 147 mV: 0.47 |
| *R*_m_ (MΩ) | 13.4 | 9.1 | 5.4 | 7.6 | 6.3 | 4.7 |
| *v*_c_: Calculated *v*_m_ | 160 mV: 133 mV | 160 mV: 136 mV | 160 mV: 149 mV | 160 mV: 117 mV | 160 mV: 128 mV | 160 mV: 147 mV |

Reversal potential: V_rev_= 0 mV

*r*: compensation rate;

NOC: Number of channels;

*v*_c_: command voltage;

*v*_m_: the real command voltage added on membrane;

*p*_o_: the open probability of channels;

*R*_m_=1/(NOC**p*_o_**G*_BK_);

*G*_BK_: Single-channel conductance of BK channels is 250 pS;

Calculated *v*_m_=(*R*_m_/(*R*_m_+(1-*r*)*R*_s_))*v*_c_
