# Supplementary material for: Native Gating Behavior of Ion Channels in Neurons with Null-Deviation Modeling
Source: PLoS One. 2013 Oct 25;8(10):e77105. doi: 10.1371/journal.pone.0077105 (PMC3808363; doi:10.1371/journal.pone.0077105)
Supplement: Table S2 — Parameters of Kv3.1 channel model. (DOCX) [file pone.0077105.s005.docx]

**Table S2. Parameters of Kv3.1 channel model.**

|  | Direct | | Native | |
| --- | --- | --- | --- | --- |
| a (ms^-1^) | 0.925973 | | 1.027813 | |
| b (mV) | 35.893170 | | 35.984422 | |
| c (ms^-1^) | 0.194083 | | 0.212136 | |
| d (mV) | 279.735292 | | 187.126824 | |
| alpha (ms^-1^) | 40.558628 | | 44.050331 | |
| beta (ms^-1^) | 389.270005 | | 368.999741 | |
| Filter(Bessel) | 4 kHz | | | |
| NOC | Activation 1192 | Deactivation 1241 | Activation 1044 | Deactivation 1082 |

NOC: Number of channels.

Single-channel conductance of Kv3.1 channels is 40 pS.

Reversal potential: V_rev_=-80 mV
