# Supplementary material for: Native Gating Behavior of Ion Channels in Neurons with Null-Deviation Modeling
Source: PLoS One. 2013 Oct 25;8(10):e77105. doi: 10.1371/journal.pone.0077105 (PMC3808363; doi:10.1371/journal.pone.0077105)
Supplement: Table S3 — Parameters of the model cell composed of Nav and Kv3.1 channels. (DOCX) [file pone.0077105.s006.docx]

**Table S3. Parameters of the model cell composed of Nav and Kv3.1 channels.**

| Channels | G (nS) | V_rev_ (mV) |
| --- | --- | --- |
| Na | 215 | +55 |
| Kv | 200 | -55 |
| Leak | 0.01 | -55 |

*C*_m_: 7 pF

Rest Potential: -54.7 mV
