# Supplementary material for: Native Gating Behavior of Ion Channels in Neurons with Null-Deviation Modeling
Source: PLoS One. 2013 Oct 25;8(10):e77105. doi: 10.1371/journal.pone.0077105 (PMC3808363; doi:10.1371/journal.pone.0077105)
Supplement: Table S4 — Comparison of parameters of BK Model derived by delayed fit (at different bandwidth) and native fit (Bessel, 15.7 kHz). (DOCX) [file pone.0077105.s007.docx]

**Table S4.** **Comparison of parameters of BK Model derived by delayed fit (at different bandwidth) and native fit (Bessel, 15.7 kHz)**

| **Parameter** | **Delay 1K** | **Delay 3K** | **Delay 15.7K** | **Bessel 15.7K** |  |
| --- | --- | --- | --- | --- | --- |
| **a4 (ms^-1^)** | 0.042229 | 0.045959 | 0.066680 | 0.070511 |  |
| **b (mV)** | 32.81188 | **35.78346** | 41.03298 | 44.43735 |  |
| **c4 (ms^-1^)** | 68.30289 | 50.93056 | 95.63144 | 105.1186 |  |
| **d (mV)** | 23.68451 | 25.97927 | 22.92576 | 21.89965 |  |
| **c3 (ms^-1^)** | **0.000011** | **0.000066** | **0.000047** | 0.000499 | 123 < 25% |
| **c2 (ms^-1^)** | **0.000006** | **0.000007** | **0.000003** | 0.000074 | 123 < 50% |
| **c1 (ms^-1^)** | **0.000009** | **0.000020** | **0.000058** | 0.000406 | **123** <100% |
| **c0 (ms^-1^)** | ***0.489819*** | ***0.512815*** | 0.158086 | 0.152026 | ***123*** ≥ 100% |

kc=1.17 μM, ko=19.52 μM for all
