# Supplementary material for: Native Gating Behavior of Ion Channels in Neurons with Null-Deviation Modeling
Source: PLoS One. 2013 Oct 25;8(10):e77105. doi: 10.1371/journal.pone.0077105 (PMC3808363; doi:10.1371/journal.pone.0077105)
Supplement: Table S5 — Signal delay caused by SOF of the AXON type patch-clamp amplifier. (DOCX) [file pone.0077105.s008.docx]

**Table S5. Signal delay caused by SOF of the AXON type patch-clamp amplifier.**

| SOF=Bessel | |  | SOF=Butterworth Filter | |
| --- | --- | --- | --- | --- |
| *f*_3dB_ (kHz) | *t*_delay_ (ms) |  | *f*_3dB_ (kHz) | *t*_delay_ (ms) |
| 2 | 0.167 |  | 3 | 0.167 |
| 3 | 0.111 |  | 4 | 0.125 |
| 4 | 0.084 |  | 5 | 0.100 |
| 5 | 0.067 |  | 6 | 0.083 |
| 6 | 0.056 |  | 7 | 0.071 |
| 7 | 0.048 |  | 8 | 0.063 |
| 8 | 0.042 |  | 9 | 0.056 |
| 9 | 0.037 |  | 10 | 0.050 |
| 10 | 0.033 |  | 20 | 0.025 |
| 20 | 0.017 |  | 30 | 0.017 |
| 30 | 0.011 |  | 45 | 0.011 |
