# Supplementary material for: Native Gating Behavior of Ion Channels in Neurons with Null-Deviation Modeling
Source: PLoS One. 2013 Oct 25;8(10):e77105. doi: 10.1371/journal.pone.0077105 (PMC3808363; doi:10.1371/journal.pone.0077105)
Supplement: Note S1 — Calculation of the filter delay caused by the EPC-9. (DOCX) [file pone.0077105.s009.docx]

**Note S1. Calculation of the filter delay caused by the EPC-9.** The block diagram in Figure 1f contains a model of the signal delay resulting from (1) stimulus pathway (which is denoted by delay1), and (2) current monitor pathway (which is denoted by delay2) of the EPC-9. Generally speaking, a signal delay caused by a system in time-domain can be predicted with the system’s group delay calculated in the frequency-domain, i.e.,

$t_{\text{d}}(\omega)=-\frac{\angle H(j\omega)}{\omega}$ (1)

Where, *t*_d_ denotes the system’s group delay; *H* the transfer function; *ω* the angular frequency. Note that *t*_d_ is a function of *ω*. Therefore, the signal delay for the EPC-9 was estimated, in practice, with the maximum *t*_d_ over the pass band.

The transfer function *H* is a product of the transfer functions of the two pathways mentioned above. Both pathways consists of several cascaded stages. Although the transfer function of each stage can be obtained from Gillis (2000), we briefly described here for the sake of convenience. The flexibility of the EPC-9 is reflected in the configurable hardware. Therefore, *t*_delay_ varies under different recording conditions. Provided a set of concrete settings, a detailed frequency spectral of each pathway can be calculated according to

$H_{\text{delay1}}=H_{\text{sf}}H_{\text{vd}}$ (2)

$H_{\text{delay2}}=H_{Rf}H_{\text{F1}}H_{\text{F2}}$ (3)

Where, *H*_sf_ denotes the transfer function of SF; *H*_vd_ that of *v_cmd_* driver; *H_Rf_* that of feedback resistor; *H*_F1_ that of F1; *H*_F2_ that of F2. The detailed transfer function of each stage is listed below:

$H_{\text{vd}}=0.1$ (4)

$H_{\text{sf}}=\left\{\begin{matrix} \frac{1}{s\tau_{2}+1}, \\ \frac{1}{\left(s\tau_{2}+1\right)\left(s\tau_{10}+1\right)^{2}}, \end{matrix}\right.$ (5)

Where, *τ*_2_=2 µs, *τ*_10_=10 µs; *s*=*jω*

$H_{\text{F1}}=\frac{1}{(s^{2}/\omega_{n}^{2}+2\varsigma s/\omega_{n}+1)(s\tau+1)}$ (6)

Where, detailed values of *ω_n_* (rad/s), *ζ* and *τ* (µs) for different settings were 8.59×10^4^, 0.728, 10 (10 kHz Bessel); 3.18×10^5^, 0.668, 4 (30 kHz Bessel); 1.10×10^6^, 0.906, 1 (100 kHz Bessel); 1.84×10^5^, 0.206, 4 (High Q).

Butterworth:$H_{\text{F2}}=\frac{1}{(s^{2}/\omega_{3\text{dB}}^{2}+1.848s/\omega_{3\text{dB}}+1)(s^{2}/\omega_{3\text{dB}}^{2}+0.765s/\omega_{3\text{dB}}+1)}$ (7)

Bessel:$H_{\text{F2}}=\frac{1}{(0.488s^{2}/\omega_{3\text{dB}}^{2}+1.916s/\omega_{3\text{dB}}+1)(0.388s^{2}/\omega_{3\text{dB}}^{2}+1.241s/\omega_{3\text{dB}}+1)}$ (8)

Time-domain delay for each pathway (*t*_delay1_ or *t*_delay2_) can be calculated with the phase of *H*_delay1_ or *H*_delay2_; i.e.,

$t_{\text{delayi}}=\mathrm{argmax}\left\{-\frac{\angle H_{\text{delayi}}(j\omega)}{\omega}\right\}$ (i=1,2) (9)

In practice, we simply consider *t*_delay1_ as a double-value constant taken to be zero for “0 µs” SF-setting and 0.02 ms for “20 µs” SF-setting. But *t*_delay2_ is more complicated, which is a function of F1-setting and F2-setting. Therefore, detailed values of *t*_delay2_ for different settings should be calculated based on *H*_delay2_. In the EPC-9, there are 2 SF-settings, 4 F1-settings, and many available F2 cut-off frequencies. Figure 1g, and Table 1 show that the total delay for the EPC-9 depended heavily on the cut-off frequency of F2. A higher F2 cut-off frequency would decrease the signal delay in responding currents.
