# Supplementary material for: Native Gating Behavior of Ion Channels in Neurons with Null-Deviation Modeling
Source: PLoS One. 2013 Oct 25;8(10):e77105. doi: 10.1371/journal.pone.0077105 (PMC3808363; doi:10.1371/journal.pone.0077105)
Supplement: Note S2 — Calculation of the filter delay caused by Axon 700B. (DOCX) [file pone.0077105.s010.docx]

**Note S2. Calculation of the filter delay caused by Axon 700B.** Based on the datasheet of the AXON patch-clamp amplifier produced by Molecular Devices, there designs a variable Scaled Output Filters (SOF) in the circuit, which can be configured to a 4-pole Bessel filter or a 4-pole Butterworth filter. Since SOF is the most influential component, we simply modeled the total signal delay (*t*_delay_) for the AXON 700B in the following way:

$t_{\text{delay}}=t_{\text{SOF}}+t_{\text{ep}}$ (10)

Where, *t_ep_* is the empirical delay value for the whole amplifier excluding the SOF. For MultiClamp 700B, *t_ep_* has the approximate value of 0.06 ms. The cutoff frequency of “Bessel” SOF of 700B ranges from 2 to 30 kHz, while that of “Butterworth” SOF ranges from 3 to 45 kHz. We calculated *t*_SOF_ for typical settings over the full range (Tabel S4).
