# Supplementary material for: Native Gating Behavior of Ion Channels in Neurons with Null-Deviation Modeling
Source: PLoS One. 2013 Oct 25;8(10):e77105. doi: 10.1371/journal.pone.0077105 (PMC3808363; doi:10.1371/journal.pone.0077105)
Supplement: Note S3 — Derivation of the relationship between v c and v m in the closed-loop R s comepnsation. (DOCX) [file pone.0077105.s011.docx]

**Note S3. Derivation of the relationship between *v*_c_ and *v*_m_ in the closed-loop *R*_s_ comepnsation.** We start from the three-component cell model, and we have

$v_{m}=v_{p}\frac{Z_{m}}{R_{s}+Z_{m}}$. (11)

Where, *Z_m_* is the equivalent complex impedance of the paralleled *R_m_* and *C_m_*; i.e.,

$Z_{m}=\frac{R_{m}}{1+sR_{m}C_{m}}$. (12)

When compensated, the pipette potential is a sum of the command voltage and a signal proportional to the pipette current (*i_p_*); i.e.,

$v_{p}=v_{c}+ri_{p}R_{s}$. (13)

Applying the Ohm law, we have

$i_{p}=\frac{v_{p}}{Z_{m}}$. (14)

Combine the four simultaneous equations, we get

$v_{\text{m}}=v_{\text{c}}\frac{R_{\text{m}}}{R_{\text{m}}+\left(1-r\right)R_{\text{s}}+sR_{\text{m}}C_{\text{m}}\left(1-r\right)R_{\text{s}}}$. (15)
