# Supplementary material for: Native Gating Behavior of Ion Channels in Neurons with Null-Deviation Modeling
Source: PLoS One. 2013 Oct 25;8(10):e77105. doi: 10.1371/journal.pone.0077105 (PMC3808363; doi:10.1371/journal.pone.0077105)
Supplement: Pseudocode S1 — Emulation of Stim Filter. (DOCX) [file pone.0077105.s012.docx]

**Pseudocode S1. Emulation of Stim Filter.**

1. tau10=0.00001,tau2=0.000002,p=exp(-interval/tau10),q=exp(-interval/tau2)
2. b1=-(2*p+q),b2=(p*p+2*p*q),b3=-p*p*q,a0=b0+b1+b2+b3
3. for i = 1 to sweepnum do
4. if (isFilter) then
5. new1protocol _i,1_ = a0*protocol_i,1_
6. new1protocol _i,2_ = a0*protocol_i,2_ - b1*protocol_i,1_;
7. new1protocol _i,3_ = a0*protocol_i,3_ - b1*protocol_i,2_ - b2* protocol_i,1_
8. for j = 4 to sweeplen do
9. new1protocol _i,j_ = a0*protocol_i,j_ - b1*protocol_i,j-1_ - b2* protocol_i,j-2_ - b3*protocol_i,j-2_
10. od
11. fi
12. od
