# Supplementary material for: Native Gating Behavior of Ion Channels in Neurons with Null-Deviation Modeling
Source: PLoS One. 2013 Oct 25;8(10):e77105. doi: 10.1371/journal.pone.0077105 (PMC3808363; doi:10.1371/journal.pone.0077105)
Supplement: Pseudocode S2 — Emulation of response filter and Rs Compensation. (DOCX) [file pone.0077105.s013.docx]

**Pseudocode S2. Emulation of response filter and Rs Compensation.**

1. for i = 1 to sweepnum do
2. Po = CalcPo(new1protocol_i,1_)
3. if (isSeriResis) then
4. Rm = 1 / (Po*gk*channelnum)
5. sp=-(Rm+ResisRs*(1-ResisRatio)) / (Rm*Cm*ResisRs*(1-ResisRatio))
6. zp=exp(sp*interval)
7. lumda=(1-zp) / ( Rm+ResisRs*(1-ResisRatio))
8. new2protocol_i,1_ = Rm*lumda*new1protocol_i,1_
9. else
10. new2protocol_i,1_ = new1protocol_i,1_
11. fi
12. current_i,1_ = Po*gk*channelnum*(new2protocol_i,1_ - Vr)
13. for j = 2 to sweeplen do
14. Po = CalcPo(new2protocol_i,j-1_)
15. if (isSeriResis) then
16. Rm = 1 / (Po*gk*channelnum)
17. sp=-(Rm+ResisRs*(1-ResisRatio)) / (Rm*Cm*ResisRs*(1-ResisRatio))
18. zp=exp(sp*interval)
19. lumda=(1-zp) / ( Rm+ResisRs*(1-ResisRatio))
20. new2protocol_i,j_ = Rm*lumda*new1protocol_i,j_ +zp*new2protocol_i,j-1_
21. else
22. new2protocol_i,1_ = new1protocol_i,1_
23. fi
24. current_i,j_ = Po*gk*channelnum*(new2protocol_i,j_ - Vr)
25. od
26. if (isFilter) then
27. if (FiltType = 1) then
28. Butterworth(current_i_)
29. fi
30. if (FiltType = 2) then
31. Bessel(current_i_)
32. fi
33. fi
34. od
